# How Can Reasoned Transparency Enhance Co-Creation in Healthcare and Remedy the Pitfalls of Digitization in Doctor-Patient Relationships?

**DOI:** 10.34172/ijhpm.2020.263

**Published:** 2021-01-02

**Authors:** Vincent Mabillard, Nicolas Demartines, Gaëtan-Romain Joliat

**Affiliations:** ^1^Solvay Brussels School of Economics and Management, Université libre de Bruxelles (ULB), Brussels, Belgium.; ^2^Department of Visceral Surgery, Lausanne University Hospital CHUV, Lausanne, Switzerland.

**Keywords:** Digitization, Transparency, Co-Creation, Artificial Intelligence, Doctor-Patient Relationship

## Abstract

This article addresses transparency in the current era of digital co-creation between healthcare professionals and patients. The concept of reasoned transparency is presented as a potential tool to guide the development of digital co-creation that is rapidly growing. The aim was to reflect on how doctors can apply transparency in their daily practice, following the shift from paternalistic to more collaborative relationships. On the one hand, our contribution indicates ways to take advantage of the existing digital tools to improve efficiency and increase patient trust, including the latest trend of artificial intelligence. On the other hand, this article identifies pitfalls of digitization and proposes reasoned transparency as remedy for the challenges rose by artificial intelligence. As a result, this perspective article tackles the issue of maintaining trustful and high-quality relationships between doctors and patients, increasingly challenged by the dissemination of online information and the pressures on healthcare professionals’ accountability towards patients and the general public.

## Introduction

 What are the pitfalls of applying transparency in the medical world, and how should doctors address this issue in their daily practice? These questions are important in the current era of data sharing and full disclosure. Transparency has for some time been a buzzword in healthcare management, where openness policies are frequently proposed as the cure to governance problems.^1^ In this sense, the opacity traditionally characterizing the healthcare domain has been increasingly questioned, especially regarding quality reporting issues.^2^ This change is the result both of ethical considerations (conflicts of interest, clinical trial conditions) and economic incentives, driven by the need to improve the healthcare system’s efficiency.^3^ Moreover, transparency has been claimed in healthcare to improve clarity, to increase patient trust, and to induce better long-term outcomes by improving quality of care.^4^

 Reflection about transparency has to be extended to daily medical practice, in which doctors are advised to openly discuss treatment, medication, or the disease progress with their patients. This development is guided by the concept of patient empowerment, shifting from a paternalistic model towards the growing involvement of patients through medical explanations, informed consent, and decision sharing. It falls under the broader notion of co-creation, which implies more frequent interactions as well as the sharing of resources and responsibilities with the aim of producing more efficient and trustworthy health solutions and outcomes.^5,6^ Co-creation is understood here as a process to provide better care service and increase the perceived value of the treatment by the patients. In this regard, maximization of care quality is ensured by frequent interactions between doctors and patients, based on active collaboration rather than passive involvement.^7^

 Co-creation should be regarded as a goal as well as a result of this shift from one-way communication to increased interaction. While digitization and the recent trend of artificial intelligence (AI) offer new opportunities to enhance the patient-doctor relationship, there are also pitfalls related to this technological evolution. This perspective article questions the implementation of transparent medical practice in an increasingly digitized environment, and proposes reasoned transparency as a remedy for the challenges highlighted in both the literature and practice, including data privacy, self-medication, or trust in the patient-doctor relationship.^8^

## Transparency and Accountability in Healthcare

 The transparency movement echoes the general call for accountability. Doctors are *held to account* by their hierarchy, their patients, the general public, politicians, and the payers; at the same time, they have to *take into account* patients’ opinions and decisions. Consequently, they are subject to vertical and horizontal forms of accountability, under growing pressure from both managers and patients. In the surgical world, this transparency quest has also pushed hospitals and surgeons in several countries to closely monitor their complication rates and make them publicly available.^9^ A potential negative consequence is that patients or people outside the healthcare system consulting the raw numbers lack important elements of context that are essential before any interpretation. For example, a particular hospital or surgeon may display higher complication or mortality rates due to the type of polymorbid and frail patients treated and operated on. The need for sound explanations to foster patients’ understanding has been labelled in other contexts (government-citizen relations) as reasoned transparency.^10^ Applied to healthcare issues, this concept enables better patient choices and decisions, assuming that it will increase people’s knowledge and understanding of the functioning and actions of public health organizations and professionals.

 Transparency has been addressed in different ways, depending on the context considered. In Switzerland, a new section of the *Law on Therapeutic Products* that came into force on January 1, 2020, introduces integrity and transparency obligations for professionals prescribing medication, forcing doctors to systematically report their activities. This law aims at encouraging patients to engage more deeply with the medical community regarding their medication and treatment. Similar legislation has been adopted earlier in some countries. An example is the U.S. *Physician Payment Sunshine Act* passed in 2013 and due to be extended to physician assistants and advance practice nurses in 2021-2022. This development, in addition to being of interest to regulators and policy-makers, gives patients more precise knowledge of doctors’ financial ties with manufacturers or drug companies. This is crucial, given that patients will increasingly do research on medications and providers. Furthermore, payment disclosures are of importance as it was estimated in a 2007 study that 94% of US doctors had links with pharmaceutical companies.^11^ In addition to deepening the patient-doctor relationship, these new legal requirements help advance the debate on external pressures on conflict of interests and the general functioning of the healthcare system.

## The Effect of Digitization on Transparency and Co-Creation Practices

 In medicine access to information and to healthcare providers is key for successful transparency.^12^ Lately, this increasing need for transparency and accountability has been reinforced by the development of new technologies. In this sense, digital medicine might improve interoperable access.^12^ Regarding the relationship between doctors and industry, traceability has to be enhanced, potentially leading to more accountability. Better treatment monitoring and follow-up may be required by patients, based on the creation of an electronic medical record, which can be transmitted to other hospitals and patients themselves. Moreover, further technological developments may reduce costs and save time through co-creation practices. Vaccination certificates to be filled online, with multiple, personalized pre-determined choices, provide a good example of what can be co-created electronically, involving the patients to ease the process, and save time and money. In addition, transparency helps improve patient safety via incident reporting.^13^ However, in terms of online access to patient data and reporting, great disparities between institutional and non-hospital (family physicians, pharmacy, etc) settings still remain and should not be overlooked. In addition, transparency of data needs safeguards to protect patient confidentiality. With digitization, it is important to have systems such as blockchain or identification access management to protect the security of data.

 Co-creation is a multifaceted notion and refers to distinct processes: relationships between suppliers and customers to improve healthcare (digital) solutions, horizontal collaborations within a hospital to improve treatment, or mutual decision between patients and their physician to deliver better care quality.^14^ Here, we prefer the latter facet of co-creation since our focus is on the relationships between patients and healthcare professionals, especially doctors. Digitization has influenced the dynamics of co-creation through widespread access to the Internet and, consequently, access to large amounts of information. While doctors remain the experts regarding clinical knowledge, patients have now authority over their own personal preferences and values.^15^ As a result, the democratization of decision reflects a cultural change, which results from the “inevitably disruptive effects of citizen-empowering technological change.”^16^ Although praised in many settings, this evolution of the patient-doctor relationship, bolstered by digital technologies, is yet to be implemented in most cases.

 As one of the latest technological developments, AI tends to reinforce the ambition of fostering a patient-centered approach.^17^ In general, AI raises great expectations since it holds the potential to reduce transaction costs, to provide ever vigilant tools, to provide physicians with up-to-date information on a timely basis and, most importantly, to help reduce therapeutical errors that can happen in human clinical practice.^18^ This global enthusiasm for AI in healthcare is in line with the massive investments in the domain, reaching around $8.5 billion, including all big tech companies, insurers, startups, pharmaceutical and medical device firms.^19^ In China, more than 300 million users have registered to the leading health-management platform, called Ping An’s Good Doctor.^19^

 AI relates to multiple services, tools and layers. As a form of digital innovation, it includes facilitated collection of a wide range of patient data, expansion and further creation of datasets, accelerated development of logical capability through physical machinery and devices, and improved services to extend diagnoses, partially based on these devices.^20^ Here, we focus on the latter service layer since it is more closely linked to the patient-doctor relationship. For instance, at-home treatments are supported by smartphone applications in selected cases, and treatment may be derived from predefined algorithms.

 Explainable AI refers to the notion of understandable results of AI.^21^ This group of methods aims to render the solutions given by AI more comprehensible to humans. In medicine, explainable AI is of importance, because deep learning results often are black-box predictions that cannot be explained to clinicians. These black-box predictions lack transparency. The challenge now is to find the best AI model that can be precise and powerful enough but at the same time explainable and transparent without being too simplistic.^22^ In that sense and in the context of co-creation, explainable AI in medicine could serve as support of co-creation by contributing to the proposed concept of reasoned transparency. For example, it is of value for the clinician to know exactly which individual parameter play an important role in an AI prediction.^22^ Explainable AI is a powerful tool and means to increase transparency and trust in a co-creation model in medicine.^23^ A further challenge for the clinician will be to popularize not only the results of AI and machine learning (ML) but also the mechanism behind it. This will require pedagogical skills and specific knowledge of AI and ML.

 The example of at-home treatment using smartphone applications goes one step further than transparency since it provides patients with a decision to make by themselves, for themselves. Paradoxically, such a system could reverse the current trend of deeper patient involvement in the therapeutic relationship. It could also broaden the gap between patients and physicians should the latter be replaced by devices, designed and perceived as outperforming healthcare professionals, thereby creating an ‘automation bias.’^20^ Such a change would certainly damage the patient-doctor relationship. At the same time, it would undermine the implicit promise of healthcare systems: to exercise good judgment, partially based on the patients’ needs, to deliver high quality care. AI and ML induce other significant challenges: importance of safeguards, risk of bias, inequity, effects on patients, legal concerns, and societal issues (trust decline or decreased value of patient choice).^24^ Several methods or safeguards have been or can be proposed to respond to these abovementioned risks of AI and ML. AI results and predictions should be first and foremost accurate.^25^ It should therefore be assessed on outcomes and be proven to improve patient outcomes.^25^ In that sense, at that moment, AI should be proposed in research settings and strong evidence on outcomes should be published. Explainability of AI and mitigation of bias should be clearly emphasized.^21^ Strict regulations and legal directives should also be created and enforced. Finally, potential conflicts of interests of AI developer companies should be exposed.

 In spite of these pitfalls, AI represents a major technological advance that will definitely enrich and help the medical world. With a capacity well beyond human brain capacities, AI may soon bring undeniable help and support for the diagnosis or treatment of patients, provided it is guided by healthcare professionals to explain and contextualize plain results and to discuss the existing therapeutic options in a trusting patient-doctor relationship. This last point is crucial: the nature of AI systems should remain assistive.^26^ In this sense, human interactions should not be fully replaced by digital devices; these tools are modeled for providing support for clinicians’ decision, which should be reached through a constructive dialogue with patients.^27^ This is how we envisage co-creation of better quality care thanks to exchanges based on reasoned transparency, capitalizing on the opportunities offered by an increasingly digitized work environment.

 However, digitization, if uncontrolled, faces another challenge. It may seriously endanger the dynamic of transparency, accountability and trust in patients’ experience with healthcare. Taking a closer look, applications are a black box, raising questions as to who or what will finally *be held accountable* for decisions taken by machine processes. This points especially to the difficulty of establishing a regulatory framework. In the United Kingdom, for instance, the Information Commissioner (in charge of public transparency and data protection) ruled out the usage of an application designed by Google DeepMind, which could alert patients at risk of renal diseases.^28^ Also related to data privacy, patients may refrain from sharing their data in the absence of a clearly established surveillance body, which ensures that such data will not be used to serve commercial purposes or health insurance interests. In this regard, healthcare does not differ from other domains and policies, where transparency and accountability are regarded as key principles to overcome this problem. In most cases, de-identification techniques are also proposed to ensure confidentiality.^29^ The main challenge of transparency is probably privacy maintenance. New technologies such as blockchains or identification access management might help combining these two issues. Blockchain technology with specific encryption and protection mechanisms offers diverse layers of transparency. Moreover, identification access management permits to keep data secure. These two examples of technological developments could be seen as safeguards that could be used without precluding transparency. A major difference in the medical world though: excessive transparency endangers medical privacy, the sacred principle underlying the patient-doctor relationship.

 Although informed by medical expertise and knowledge, this new, technological-oriented approach to treatment will not solve a problem already faced by doctors and patients discussing therapeutic options: technical issues remain better understood by specialists, who can then provide contextualized advice. In this sense, even though co-creation practices will certainly turn more digital with the development of AI, patients will still have a deficit of information, which can lead to dramatic consequences (this is already the case, for example, when people prefer to self-medicate based solely on information retrieved online).

 AI is expected to overcome this last challenge by providing timely and accurate answers to people through algorithms. Consequently, this process may disrupt the exchanges between doctors and patients, currently positively evolving from one-way communication to active involvement of patients in their care. For example, treatment options following genetic testing have to be discussed and decisions have to be taken in concert with the patient. Through a detailed discussion, information can be shared more deeply. Such reasoned transparency may be seriously damaged by the dictatorship of algorithms, thus unraveling current efforts to establish more qualitative relationships between medical staff and patients, including personal feelings and psychological monitoring. Conversely, processing of information thanks to AI may allow additional time to physicians, which can be spent to have fruitful discussions with the patients, enabling doctors to better understand the patients’ values and deliver individualized care in a better way.^30^ In this vein, digital tools, including AI devices, and reasoned transparency do not seem to be automatically at odds, and may well lead to trustful relationships between patients and their doctors, sustained by mutually beneficial co-creation practices.

## Conclusion

 In conclusion, reasoned transparency should therefore be the concept to develop in the near future to avoid the pitfalls of the upcoming digitization of healthcare. This points to the necessity of empowering patients through a strategy of risk-benefit communication, including the opportunities and limitations of digital applications for patient treatment. Moreover, doctors should endeavor to contextualize all results and treatments since most digital tools, and AI in particular, do not explain the recommendations made. Consequently, trust may be eroded or compromised due to the potential clash between treatment recommendations online, physician judgment and patient autonomy of decision.^31^ Therefore, reasoned transparency invites physicians to communicate abundantly about the usage of digital tools and devices, reassure patients about data confidentiality, increase patient knowledge about the treatment, and ensure a favorable environment to foster co-creation practices.

 All told, the current fascination with AI, which holds out great potential while giving the illusion of full transparency, must not undermine the long-standing bonds of trust between healthcare professionals and patients, which should remain the cornerstone of the therapeutic relationship. This point seems even more important in our era, characterized by an increasing reliance on technology, performance and online information/tools, no matter how misleading.

## Ethical issues

 Not applicable.

## Competing interests

 Authors declare that they have no competing interests.

## Authors’ contributions

 VM: concept and design of the article, drafting of the manuscript, acceptance of final version. ND: concept and design of the article, critical review of the manuscript, acceptance of final version. GRJ: concept and design of the article, drafting of the manuscript, acceptance of final version.
